# Host Association and Spatial Proximity Shape but Do Not Constrain Population Structure in the Mutualistic Symbiont Xenorhabdus bovienii

**DOI:** 10.1128/mbio.00434-23

**Published:** 2023-05-08

**Authors:** Bhavya Papudeshi, Douglas B. Rusch, David VanInsberghe, Curtis M. Lively, Robert A. Edwards, Farrah Bashey

**Affiliations:** a Flinders Accelerator for Microbiome Exploration, Flinders University, Adelaide, Australia; b National Centre for Genome Analysis Support, Pervasive Institute of Technology, Indiana University, Bloomington, Indiana, USA; c Center for Genomics and Bioinformatics, Indiana University, Bloomington, Indiana, USA; d Department of Biology, Emory University, Atlanta, Georgia, USA; e Department of Biology, Indiana University, Bloomington, Indiana, USA; Department of Veterinary Medicine

**Keywords:** symbiont, generalists, population structure, host specificity, spatial structuring, host-microbe

## Abstract

To what extent are generalist species cohesive evolutionary units rather than a compilation of recently diverged lineages? We examine this question in the context of host specificity and geographic structure in the insect pathogen and nematode mutualist Xenorhabdus bovienii. This bacterial species partners with multiple nematode species across two clades in the genus Steinernema. We sequenced the genomes of 42 X. bovienii strains isolated from four different nematode species and three field sites within a 240-km^2^ region and compared them to globally available reference genomes. We hypothesized that X. bovienii would comprise several host-specific lineages, such that bacterial and nematode phylogenies would be largely congruent. Alternatively, we hypothesized that spatial proximity might be a dominant signal, as increasing geographic distance might lower shared selective pressures and opportunities for gene flow. We found partial support for both hypotheses. Isolates clustered largely by nematode host species but did not strictly match the nematode phylogeny, indicating that shifts in symbiont associations across nematode species and clades have occurred. Furthermore, both genetic similarity and gene flow decreased with geographic distance across nematode species, suggesting differentiation and constraints on gene flow across both factors, although no absolute barriers to gene flow were observed across the regional isolates. Several genes associated with biotic interactions were found to be undergoing selective sweeps within this regional population. The interactions included several insect toxins and genes implicated in microbial competition. Thus, gene flow maintains cohesiveness across host associations in this symbiont and may facilitate adaptive responses to a multipartite selective environment.

## INTRODUCTION

Microbes live in complex and abstract microenvironments, obscuring our ability to determine what evolutionary forces structure the diversity we observe. Additionally, it is challenging to predict *a priori* the extent to which closely related isolates sampled from a specific region or habitat reflect a cohesive unit, distinct from other such units. As in macroorganisms, genetic distance can increase with geographic distance within microbial species (1–4) and be correlated with distinct habitats (5, 6), indicating that homogenizing forces (i.e., selection, drift, and gene flow) are more likely to operate with physical and ecological proximity. However, diverse population structures are observed across bacterial species. For instance, nearly identical isolates of Staphylococcus aureus and Vibrio cholerae have been found globally (7–9), while in other species, sympatric isolates are found to be genetically differentiated and nonrecombining (1, 10, 11), demonstrating that divergence can arise and be maintained at a small spatial scale. A key goal remains to link geographic patterns to the evolutionary forces shaping microbial populations.

Work on host-associated microbes has examined the role of hosts in governing the population structures of their symbionts. Some host specialist pathogens, such as Mycobacterium tuberculosis, display a long history of coevolution that can be seen by congruent phylogenies between the pathogens and their human host populations (12), while others, such as Helicobacter pylori, reflect more recent human migrations (13). In contrast, host generalists, such as Campylobacter species and Escherichia coli, show little signature of host species association (14) and are found to be structured more by geography than host phylogeny (15, 16). However, within some host generalist species, lineages can be found that are host specific and contain niche-adaptive genes (17–19). While most research examining population structure has been done on pathogen species of human health or economic concern, it is important to study diverse species to better understand the processes shaping microbial evolution (20).

Among beneficial symbionts, a range of population structures has also been observed. The well-studied mutualist Vibrio fisheri, associated with Hawaiian bobtail squid, shows little geographic structure or specificity to genetically distinct host populations (21). In contrast, vertically transmitted symbionts like Buchnerna aphidocola show structuring across aphid species and with host geography (22). Symbiont population structure can also be affected by host ecology (23). For example, the ant mutualist Pseudonocardia actinobacteria shows kilometer-scale geographic structuring within a single ant species that is correlated with its ability to inhibit a virulent fungal pathogen of its host (24). Here, we examine the population structure of Xenorhabdus bovienii, a mutualistic symbiont of nematodes and a virulent insect pathogen, in a region where multiple nematode species occur in sympatry.

**FIG 1:**
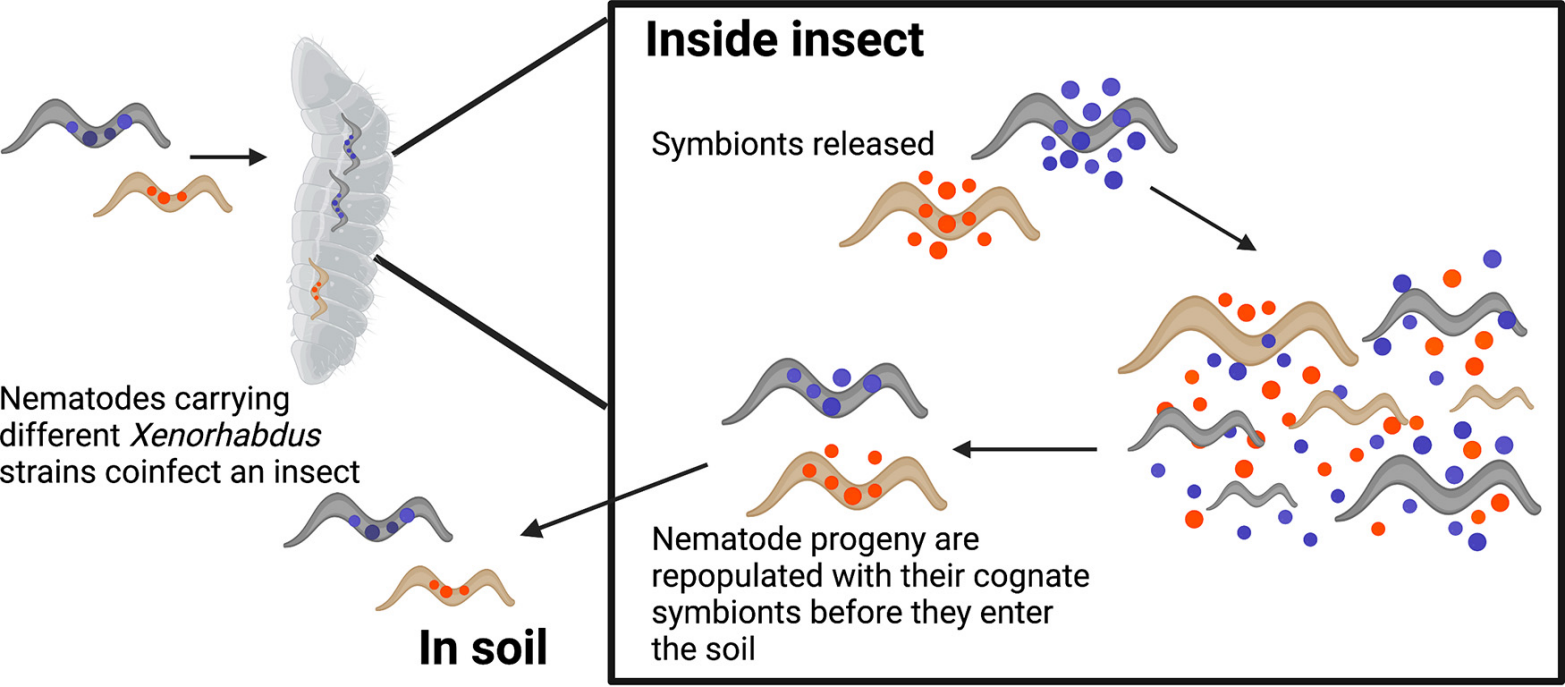
Representation of the Xenorhabdus-Steinernema life cycle. Nematodes carrying different Xenorhabdus symbionts co-occur in the soil and coinfect an insect host. Inside the insect, nematodes release their symbionts, which replicate and produce toxins, killing the insect. The nematodes also replicate for one or more generations, producing offspring that do not carry the symbionts. When resources within the insect are depleted, nematode offspring reassociate with their cognate symbionts and nematode-symbiont pairings emerge into the soil. This image was generated using BioRender.

The bacterial genus Xenorhabdus is exclusively found associated with nematodes in the genus Steinernema. These nematodes depend on Xenorhabdus for successful colonization and reproduction within insect hosts (Fig. 1), while Xenorhabdus relies on the nematodes for survival and access to insects (25). Across the genera, there is a partial congruence between the host and symbiont phylogenies, with both cospeciation and host switching observed (26). X. bovienii is noted within the genus for its ability to associate with multiple nematode species across two distinct clades of Steinernema nematodes (26). Despite this broad host range, partial cocladogenesis between X. bovienii and its nematode partners suggests specialization (27). Furthermore, experimental pairings demonstrate that the fitness of both partners declines with phylogenetic distance from native association (27–30). So, while on one hand there is evidence that X. bovienii can coevolve to form specialized partnerships, on the other hand, there is evidence that this species can be considered a host generalist (26, 27). To reconcile these findings, we sequenced genomes of X. bovienii isolated from four nematode host species across three study sites and compared them to all available genomes of this species. We hypothesized that this host generalist symbiont would comprise multiple, largely host-specific lineages and sought to identify genetic markers of such specificity. Additionally, we hypothesized that spatial proximity would facilitate genetic similarity via shared selective pressures, neutral processes, and gene flow, and thus, we tested for evidence of recent gene flow among the isolates and whether gene flow and genetic similarity were limited by host species or geographic distance.

## RESULTS

### Overview of *X. bovienii* genomes collected from Indiana.

**FIG 2:**
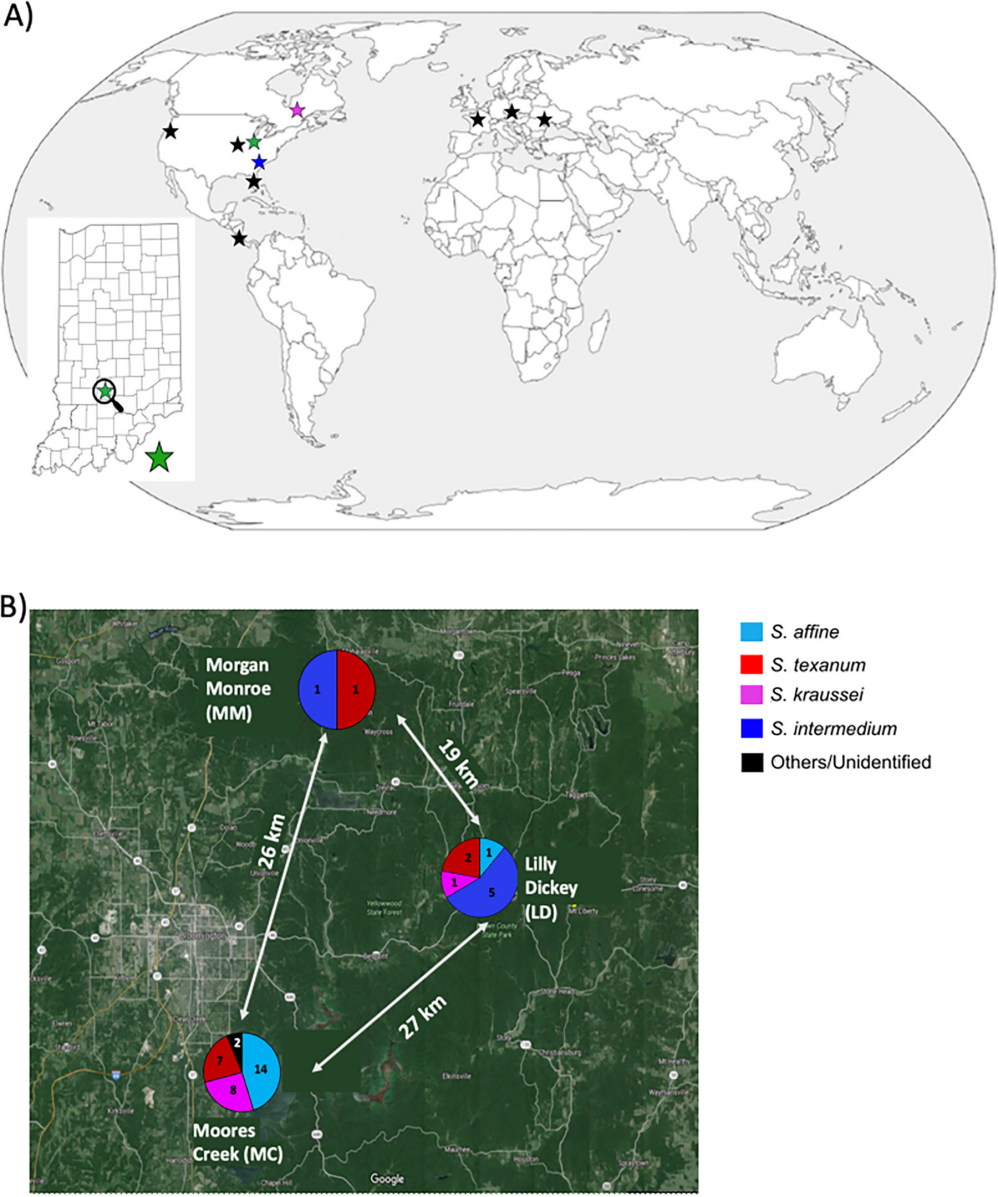
Sample distribution of Xenorhabdus bovienii genomes. (A) Newly sequenced isolates were collected from Indiana, USA, as depicted with a green star, while reference genomes from other studies deposited to NCBI and downloaded for this paper are represented in stars color coded based on the nematode host and collection. (B) Indiana isolates analyzed in this paper were collected from three Indiana University Research and Teaching Preserve sites within a 240-km^2^ region. Pie charts depict the relative numbers of isolates collected at each site and their nematode host associations. See Table S1 and NCBI BioProject accession number PRJNA700777 for information on each genome. Map outline and snapshot from Google Maps.

Genomes were obtained from 42 X. bovienii field isolates from four nematode host species across three study sites in a 240-km^2^ region of Indiana (Fig. 2). Each genome sequenced had an average of 42× genome coverage and was assembled to an average of 555 contigs. The least fragmented genome was 113 contigs (*N*_50_, 183,901 bp; total length, 4.56 Mbp), and the most fragmented was 4,329 contigs (*N*_50_, 42,376 bp; total length, 6.35 Mbp). On average 4,151 ± 312 proteins (mean ± standard deviation) were identified per isolate (range, 3,687 to 5,014), with 31.11% of the proteins annotated as hypothetical proteins. Comparisons of these genomes with 11 X. bovienii reference genomes, four Xenorhabdus nematophila genomes, and four Photorhabdus genomes show that all X. bovienii genomes have high nucleotide similarity (>94% average nucleotide identity [ANI], Fig. S1) and form a monophyletic group based on a phylogeny of the core genome (100% support), distinct from other entomopathogenic bacteria (Fig. S2 in the supplemental material).

10.1128/mbio.00434-23.4 TABLE S1 Bacterial genomes and nematode genes along with NCBI accession numbers used in this paper. Download Table S1, XLS file, 0.02 MB. Copyright © 2023 Papudeshi et al. 2023 Papudeshi et al. https://creativecommons.org/licenses/by/4.0/ This content is distributed under the terms of the Creative Commons Attribution 4.0 International license.

### Regional *X. bovienii* isolates form two distinct lineages partially based on nematode hosts.

**FIG 3:**
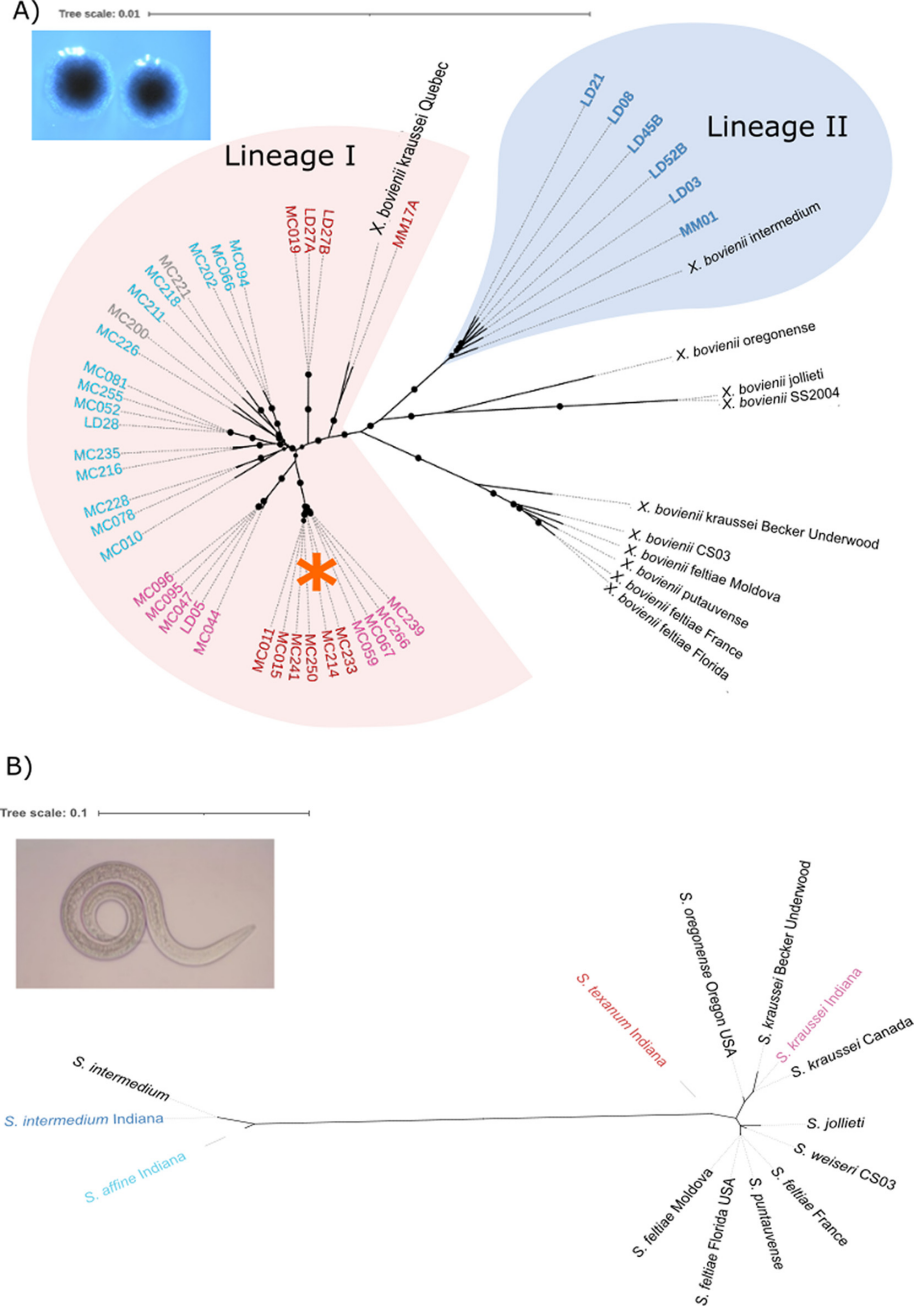
(A) Bacterial phylogeny with an image of X. bovienii colonies. Phylogenetic tree built using core genes from 53 X. bovienii genomes, with the reference genomes shown in black, the 42 Indiana isolates color coded based on the nematode host, and two samples in gray that were isolated from an unidentified nematode host. The circles represent branches with bootstrap values between 80% and 100%. The tree was built using RAxML based on the alignment of 2.18 Mb. Branch lengths have been corrected for recombination using ClonalFrameML. The orange asterisk represents bacterial isolates from two nematode species that form a monophyletic group. (B) Nematode phylogeny with an image of a Steinernema nematode. The nematode phylogeny was built from aligning 653 bp of the 28S rRNA gene using the general time reversible model of the maximum-likelihood method in MEGA. The nematode species are color coded and named to match their corresponding symbionts across the two trees.

Alignment of the 53 available X. bovienii genomes (42 Indiana isolates and 11 reference genomes), results in a core region of 2,176,418 bp, which is 45.25% of the mean genome size. Phylogenetic analysis based on this alignment shows that 36 of the Indiana isolates group with reference genome X. bovienii strain kraussei Quebec forming lineage I (Fig. 3A). Lineage I comprises all of the isolates associated with three of the nematode species: Steinernema kraussei, Steinernema texanum, and Steinernema affine. The remaining six Indiana isolates are all associated with Steinernema intermedium nematodes (Fig. 3A, dark blue labels), and they form a monophyletic group (lineage II) with the reference X. bovienii strain intermedium. Thus, the bacterial phylogeny (Fig. 3A) is not congruent with the nematode phylogeny (Fig. 3B), where S. intermedium and S. affine group together, while S. kraussei and S. texanum belong to another clade. Notably, while all of the isolates from the Moore’s Creek (MC) site are members of lineage I, isolates from the other two sites are found in both lineage I and II and cluster according to the nematode host.

Cophylogenetic analysis using Parafit shows a nonrandom association between nematode species and X. bovienii isolates (ParafitGlobal = 0.003, *P* value = 0.001), supporting the clustering based on nematode host seen in Fig. 3A. Nevertheless, this clustering is only partial. While all of the isolates associated with S. affine form one distinct group (Fig. 3A, light blue labels), the other two species in lineage I do not. Isolates associated with S. kraussei form two distinct, well supported clades (Fig. 3A, pink labels) and do not form a monophyletic group with either of the two reference genomes associated with S. kraussei. Similarly, isolates from S. texanum form three distinct, well supported clades (Fig. 3A, red labels). Thus, host switching likely has occurred in this mutualism, as indicated by maximum-parsimony reconciliation (Fig. S3).

10.1128/mbio.00434-23.1 FIG S1 Average nucleotide identity (ANI) of the whole genomes were compared across all 61 genomes that includes 42 *X. bovienii* Indiana isolates, and 11 reference *X. bovienii* genomes, 4 X. *nematophila* spp and 4 Photorhabdus spp using FastANI (https://github.com/ParBLiSS/FastANI.git). Hierarchical clustering was performed on the Euclidean distance tables. The heatmap show genome similarity, ranging from approximately 80% ANI in blue to 100% ANI in red. This figure shows *Photorhabdus* spp are equally distant to the two *Xenorhabdus* species, and that *X. nematophila* show an average of 82% ANI with *X. bovienii*. Similarity between *X. bovienii* isolates ranges from 94.34–99.99%. The Indiana isolates had a minimum of 96.9 % ANI and clustered into two distinct groups of more than 98% similarity. The first group includes 36 isolates plus the reference *X. bovienii* kraussei Quebec. The remaining six Indiana isolates were grouped with reference genome *X. bovienii* intermedium. Finally, the remaining nine reference *X. bovienii* genomes clustered together averaging 96.3% ANI. Download FIG S1, TIFF file, 1.7 MB. Copyright © 2023 Papudeshi et al. 2023 Papudeshi et al. https://creativecommons.org/licenses/by/4.0/ This content is distributed under the terms of the Creative Commons Attribution 4.0 International license.

**FIG 4:**
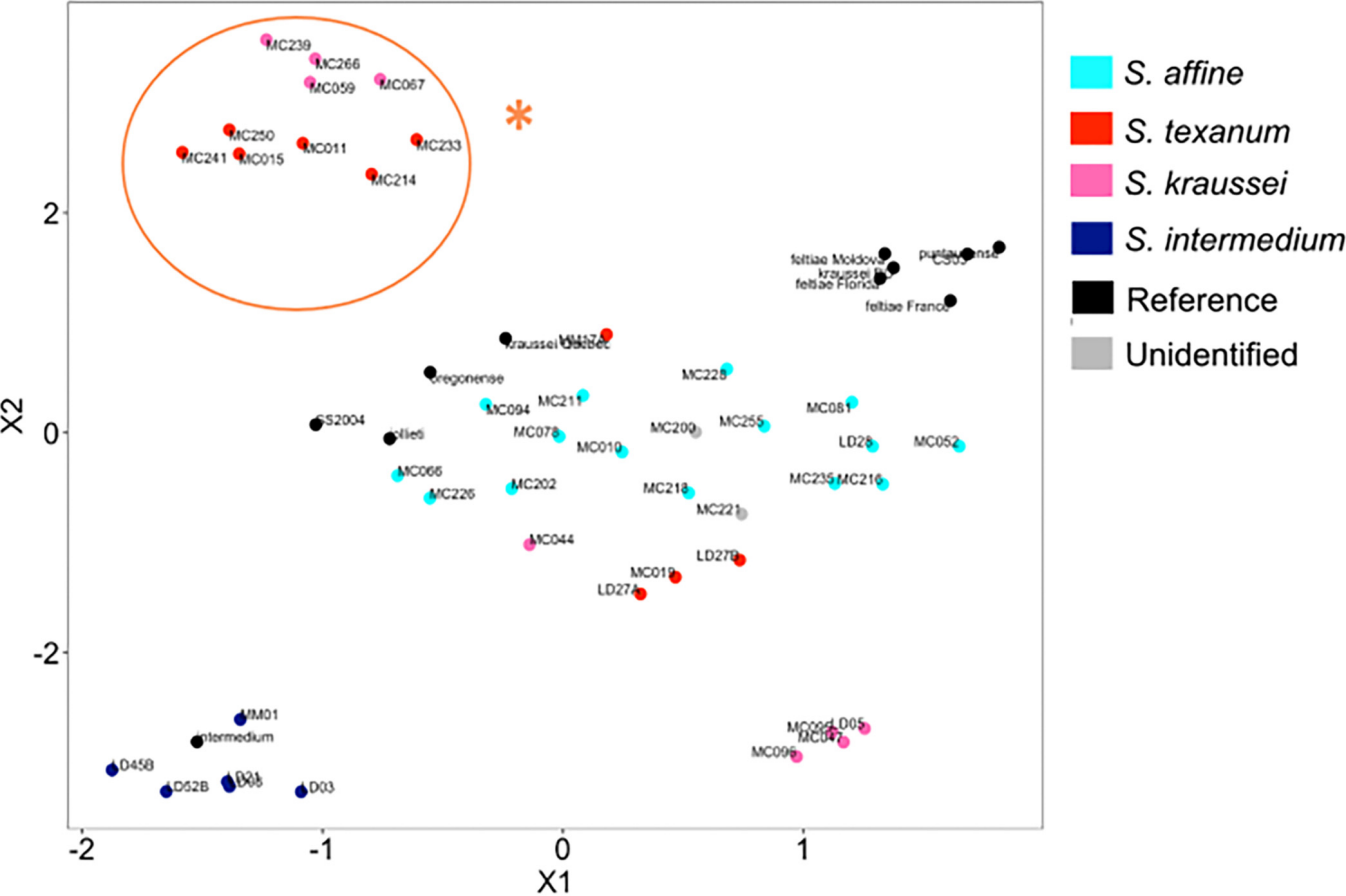
UMAP visualization based on gene presence and absence in the flexible gene set, with each data point representing a genome that is color coded based on the nematode host. The orange asterisk shows isolates from two nematode species that form a monophyletic group in the core phylogeny shown in Fig. 3B and also form a distinct cluster based on the flexible genome.

Examination of the flexible gene content shows a pattern similar to that of the core phylogeny. Roary identified 2,147 genes as core and 15,867 genes as flexible in the X. bovienii pangenome. Clustering based on gene presence and absence places the X. bovienii isolates from S. intermedium (lineage II in Figure 3A, labeled in dark blue) in a distinct part of uniform manifold approximation and projection (UMAP) space (Fig. 4, bottom left corner). Lineage I isolates fall along the diagonal, with S. affine-associated isolates (Fig. 4, light blue labels) found more centrally, while isolates from S. kraussei and S. texanum are more dispersed. Additionally, the isolates in the monophyletic clade associated with S. kraussei and S. texanum (Fig. 3A, labeled with an asterisk) form their own distinct cluster (Fig. 4, labeled with an asterisk). Thus, both the core and flexible genes support partial clustering based on the nematode host species, with isolates associated with S. kraussei and S. texanum showing recent host shift or gene exchange.

### Gene association testing across nematode hosts.

To determine if any genetic markers could predict nematode host species association, we tested the null model that single nucleotide polymorphisms (SNPs) in the core genome or flexible genes are randomly associated with respect to nematode host species by using treeWAS on all 53 X. bovienii genomes. Only one host, S. texanum, showed any significant deviations from the null model, with eight significant genes from the flexible genome. These eight genes were annotated as a putative invasin gene, a colocalized four-gene restriction modification system, a transposase gene, and two hypothetical protein genes. To determine whether this result was sensitive to genes in the global isolates, we repeated the analysis using only the Indiana isolates. Again, only S. texanum-associated isolates showed any significant genetic markers (Table S2).

10.1128/mbio.00434-23.5 TABLE S2 Host-specific genetic markers for each of the four nematode hosts, listed by whether the marker is in the core (number of unique SNPs) or flexible (number of unique genes) genome. The first number given is from the analysis of 53 *X. bovienii* isolates, and the second number is from the analysis of only the 42 regional isolates. Markers that were found to be statistically significant using treeWAS (*P* < 0.05) are indicated with an asterisk. Download Table S2, DOCX file, 0.03 MB. Copyright © 2023 Papudeshi et al. 2023 Papudeshi et al. https://creativecommons.org/licenses/by/4.0/ This content is distributed under the terms of the Creative Commons Attribution 4.0 International license.

### Indiana isolates share recent gene flow.

Homologous recombination among all the available X. bovienii genomes was assessed on the core genome using ClonalFrameML. For the 53 X. bovienii genomes, recombination rate (R) was half the mutation rate (θ), such that R/θ = 0.49. Although, recombination (r) had twice the effect on the core genome as mutation (m), as the average length of the recombination fragments was estimated as 200 bp, such that *r*/*m* = 2.49. Removing the global reference genomes showed similar results. Among the 42 Indiana isolates, recombination was more frequent (*R*/θ = 0.57) but had a similar effect on the genome (*r*/*m* = 2.37), as the length of the recombined fragments was slightly smaller (length of recombined fragment = 167 bp). Thus, the relative impact of homologous recombination in X. bovienii was similar to that found in other terrestrial gammaproteobacteria (31).

**FIG 5:**
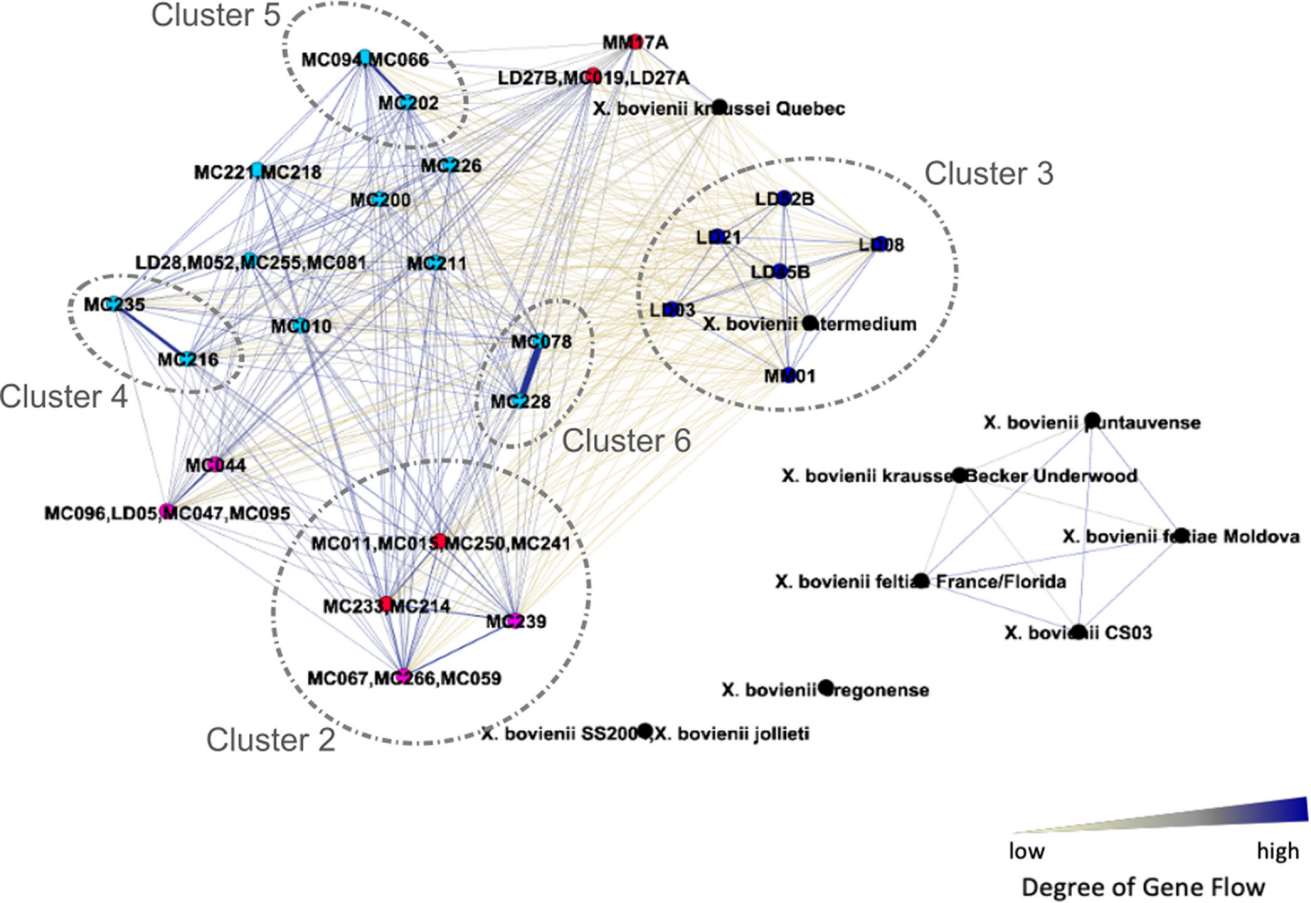
Connectivity among the 53 X. bovienii isolates shows that all Indiana isolates shared gene flow with each other and with two of the reference genomes, X. bovienii intermedium and X. bovienii kraussei Quebec, while the rest of the reference genomes formed three distinct populations with no gene flow among them. Within the Indiana population, six subclusters based on relative gene flow were identified. Nodes represent the genomes and are color coded based on the nematode host (light blue, S. affine; pink, S. kraussei; dark blue, S. intermedium; and red, S. texanum), while edges represent the degree of gene flow, with the lighter/thinner edges having lower gene flow than the darker/thicker edges. Some nodes represent multiple isolates, which are identified as clonal. Furthermore, cluster 1 is not labeled, as it was deemed to be a catchall cluster.

To better understand the extent to which gene flow is impacting the evolution of X. bovienii, we employed the recombination-based clustering analysis tool PopCOGenT on the 53 X. bovienii isolates. In contrast to ClonalFrameML, which identifies recombination in only the core genes, PopCOGenT uses pairwise alignments to test for recent genetic exchange in both the core and flexible regions and then applies network analysis to group isolates that share such exchanges. Four distinct populations with no gene flow between them were identified (Fig. 5). Three of these populations consisted of only reference genomes. Notably, the fourth population consisted of the 42 Indiana isolates along with the reference genomes of X. bovienii strain intermedium (isolated from South Carolina, USA) and X. bovienii kraussei Quebec (isolated from Canada). Thus, recent gene flow was found to connect all of the Indiana isolates.

Focusing on the Indiana isolates, we found that some isolates shared more gene flow among them than others, such that six distinct clusters were identified (Fig. 5). Cluster 1 is the largest cluster, including 19 genomes with isolates from three of the nematode hosts. Cluster 2 includes 10 genomes and is noteworthy as it comprises isolates from two nematode hosts, S. kraussei and S. texanum. This population cluster is also distinct in the phylogenetic and pangenomic analyses (Fig. 3A and 4, marked with an asterisk in each). Cluster 3 consists of six isolates, which are all the isolates associated with S. intermedium. This cluster is also consistent with the grouping observed in phylogenetic and pangenomic analyses, forming its own distinct group (Fig. 3A and 4). Clusters 4, 5, and 6, each consisting of two or three isolates, show the highest levels of gene flow and are from the nematode host S. affine.

10.1128/mbio.00434-23.2 FIG S2 Phylogenetic tree based on the core genes identified from all 61 genomes including *Photorhabdus* spp (outgroup), *X. nematophila*, reference *X. bovienii*, and 42 *X. bovienii* Indiana isolates (in bold). The tree was built from an alignment of 273 kb using GTRGAMMA model in RAxML with bootstrapping, and the extended majority rule was used to build the above consensus tree. Download FIG S2, TIFF file, 3.4 MB. Copyright © 2023 Papudeshi et al. 2023 Papudeshi et al. https://creativecommons.org/licenses/by/4.0/ This content is distributed under the terms of the Creative Commons Attribution 4.0 International license.

### Differential selection within the Indiana population.

PopCOGenT identifies genomic regions under selection by finding distinct genetic changes across clusters that show low nucleotide diversity within each cluster, suggesting a recent selective benefit or gene-specific selective sweep (32). These gene sweeps may provide insights into the traits that are adaptive in this population. For cluster 1, only one gene sweep of 1,940 bp in length (includes two genes annotated as hypothetical protein and acetyltransferase genes) was identified within the core genes, and no flexible gene sweeps were identified. Although this cluster contains the largest number of genomes, the small number of genes identified as possibly under selection suggests that this is a catchall cluster. This cluster reflects gene flow among isolates but does not show selective divergence. On the other end of the spectrum, clusters 4, 5, and 6 include fewer than three genomes each, too few to infer that recently shared genomic regions reflect selection.

In cluster 2, which contains isolates associated with two nematode host species, we identified 37 gene sweeps within the core regions, with a total length of 83.2 kb, and 34 flexible genes being swept (Table 1). The genes included encoded several insect toxins, antibiotics, and nonribosomal peptide synthetases (NRPS), as well as genes conferring resistance and stress tolerance and involved in motility, biosynthesis, and transport. Similar categories of genes were identified as showing evidence of selection in cluster 3, which includes all six isolates from the nematode host S. intermedium. Additionally, genes associated with type VI secretion systems (T6SSs), siderophore (pyochelin) biosynthesis, the *Mrx* fimbria region, and involved in iron transport were found to be sweeping through this cluster for a total of 40 sweeps with a total length of 117 kb in the core region and 66 flexible gene sweeps (Table 2).

**TABLE 1:** Summary of gene sweeps across population cluster 2, which includes 10 isolates from nematode hosts S. kraussei and S. texanum, within the core and flexible genes^*a*^

Function	Core gene sweeps (*n* = 37)		Flexible gene sweeps (*n* = 34)	
	No. of genes	Gene products or functions	No. of genes	Gene product(s) or functions
Toxin	5	RtxA, Tc	1	Tc
Nonribosomal peptide synthetase	7		1	
Antimicrobial			4	Phenazine, validamycin
Resistance	1	tellurium		
Tolerance	5	DNA repair, damage-inducible protein		
Motility	2	Fimbriae, flagella		
Regulation	5	Transcriptional, translational		
Transport proteins	2	Amino acid		
Catabolic	5	Carbohydrate, fatty acid, aminopeptide, protein	3	Carbohydrate, ATP
Biosynthesis	11	Heme, fatty acid, histidine, molybdopterin, phenylalanine, ornithine, vitamin K2, vitamin B12	1	Vitamin B6
Mobile element			8	Phage, IS
Hypothetical			16	

a For analysis of the core alignment, the sweep regions identified can include multiple genes, so the total number of genes in the table is higher than the number of sweep regions. More information about each sweep can be found in Tables S3 and S4.

**TABLE 2:** Summary of gene sweeps across population cluster 3, which includes all isolates from the nematode host S. intermedium^*a*^

Function	Core gene sweeps (*n* = 40)		Flexible gene sweeps (*n* = 66)	
	No. of genes	Gene products or functions	No. of genes	Gene products or functions
Toxin	2	RtxA, Tc	1	Hemolysin
Nonribosomal peptide synthetase	3			
Antimicrobial/anti-immune	2	NRPS dependent, membrane/LPS	18	Type VI secretion system
Resistance	8	Multidrug transports, tellurium, bleomycin, streptomycin	2	AMP, phage
Tolerance	3	DNA repair, persistence, damage inducible protein	4	DNA repair, stress response
Motility	2	Fimbriae related, oxygen sensor motility response		
Regulation	2	Transcriptional, translational		
Transport proteins	8	Siderophore, peptide, purine, zinc, potassium	6	Iron, amino acid, pigment
Catabolic	6	Lipase, phenylacetic, arginine, glycolysis, phosphatase	2	glycolysis
Biosynthesis	6	Alkaloid, heme, amino acid, folate	9	Siderophore, peptide
Hypothetical	2		24	

a For analysis of the core alignment, sweep regions, which can include multiple genes, are identified, so the total number of genes in the table is higher than the number of sweep regions. More information about each sweep can be found in Tables S3 and S4.

10.1128/mbio.00434-23.6 TABLE S3 Core gene sweeps identified in two PopCOGenT population clusters, clusters 2 and 3. Highlighted rows mark the genes that are found in both clusters. Download Table S3, XLS file, 0.04 MB. Copyright © 2023 Papudeshi et al. 2023 Papudeshi et al. https://creativecommons.org/licenses/by/4.0/ This content is distributed under the terms of the Creative Commons Attribution 4.0 International license.

10.1128/mbio.00434-23.7 TABLE S4 Flexible gene sweeps across clusters 2 and 3 from PopCOGenT clusters. Download Table S4, XLS file, 0.2 MB. Copyright © 2023 Papudeshi et al. 2023 Papudeshi et al. https://creativecommons.org/licenses/by/4.0/ This content is distributed under the terms of the Creative Commons Attribution 4.0 International license.

**FIG 6:**
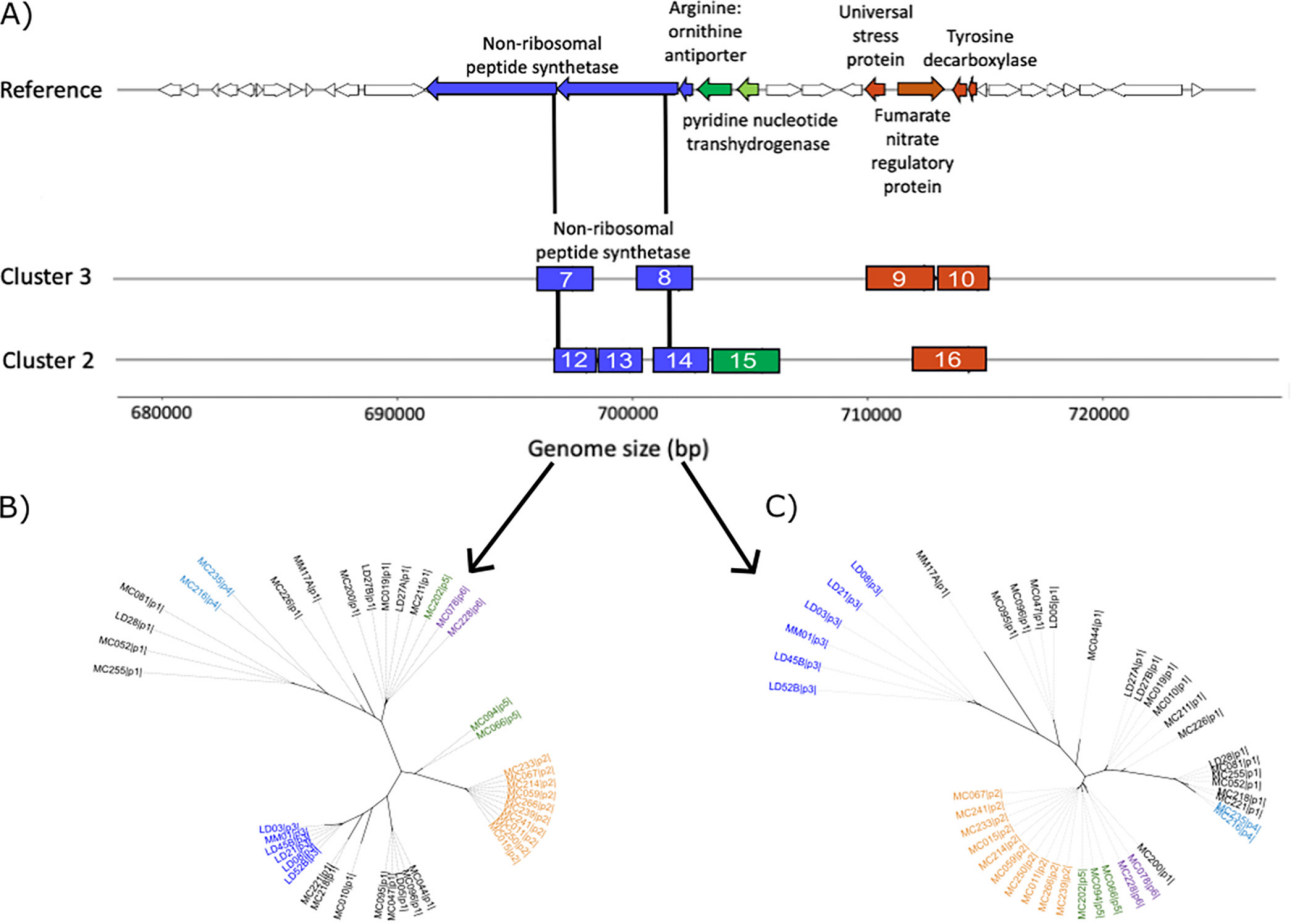
Differential gene sweeps occurring in the same region of the core genome. (A) Top, genes between 680 kb to 720 kb in the reference genome represented with block arrows showing their orientation; bottom, sweeps identified in population clusters 2 and 3 are shown as boxes. The numbers in the boxes (gene sweeps) correspond to the gene sweep identification number (ID) given by PopCOGenT analysis (Table S3). Sweep regions are unique to each cluster and identified by low nucleotide diversity; they can include only part of a gene or several genes. (B) Tree of the nonribosomal peptide synthetase region (gene sweep ID 7 in cluster 3 and gene sweep ID 12 in cluster 2) showing differentiation across clusters 2, 3, 4, and 6. (C) Tree of another nonribosomal peptide synthetase sweep region (gene sweep ID 8 in cluster 3 and gene sweep ID 14 in cluster 2) showing differentiation across clusters 2 to 6. Across the two trees, cluster 2 is highlighted in orange, cluster 3 in dark blue, cluster 4 in light blue, cluster 5 in green, and cluster 6 in purple.

Gene sweeps are by definition unique to each cluster; however, in eight cases, the same region is being differentially selected across clusters (Table S3). For instance, a 30-kb region spanning from kilobase 680 to kilobase 720 (Fig. 6A) encompasses three of these genes (2 NRPS genes and *fnr*). An examination of the corresponding gene trees shows that sometimes additional clusters are segregating at these regions as well. Specifically, an NRPS gene at kilobase 696 separates clusters 2, 3, 4, and 6 into monophyletic groups (Fig. 6B), while the neighboring gene (kilobase 701) shows that clusters 2 to 6 are all distinct (Fig. 6C). Other regions in the core alignment showing differential sweeps contain toxin genes (*Tc* and *rtxA*), regulatory genes (hrpA and azoR), a gene conferring tellurium resistance, and a dihydrolipoyl dehydrogenase gene.

### Spatial proximity and shared nematode host shape population structure.

**FIG 7:**
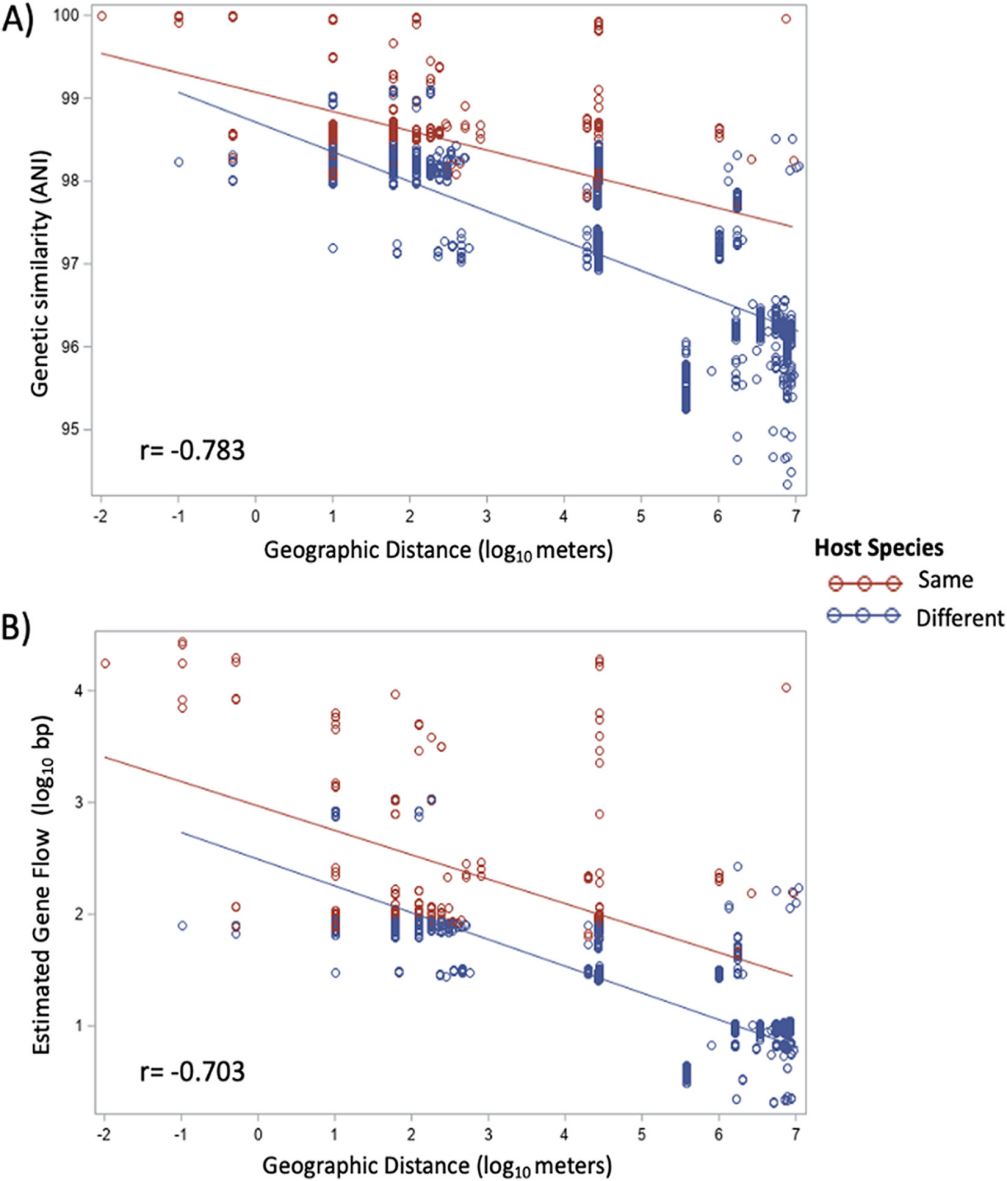
(A) Genetic similarity (ANI) and (B) gene flow (estimated length bias from PopCoGenT) decrease with geographic distance (in log meters) across all 52 X. bovienii genomes. Each point represents a pair of X. bovienii isolates and is coded by whether they were isolated from the same nematode host species (red) or from two different species (blue). Correlations across all pairs are shown in the left corner of each graph and were found to be significant with Mantel tests (*P* = 0.001). Mixed-model analyses of covariance show that both genetic similarity (*F*_1,1131_ = 30.33, *P* < 0.0001) and gene flow (*F*_1,1131_ = 78.20, *P* < 0.0001) are significantly higher if isolates are from the same nematode host species than if they are from different host species.

Despite the somewhat loose association between X. bovienii and its nematode hosts shown in the above-described analyses, mixed-model analyses of covariance (ANCOVAs) show that isolates share higher genetic similarity (*F*_1,1131_ = 30.33, *P* < 0.001) and estimated gene flow (*F*_1,1131_ = 78.20, *P* < 0.001) if they are associated with the same nematode host species (Fig. 7, Table S5). Moreover, both genetic similarity and gene flow between isolates decline significantly with distance, which ranges from 1 cm to 800 m for isolates collected within the same site, to 28 km across sites within Indiana, and up to 10 Mm with the reference sequences (Fig. 7, Mantel tests given in Table S5). These findings remain significant if the analysis is restricted to all 42 Indiana isolates or just the MC or LD isolates (Table S5).

10.1128/mbio.00434-23.3 FIG S3 Cophyletic analysis between X. bovienii and its nematode hosts. (A) Possible maximum parsimony reconciliations between the 53 X. bovienii phylogeny and its nematode hosts as estimated by eMPRess. Reconciliations vary depending on the relative costs of different evolutionary events. For each region of the parameter space, the estimated number of cospeciation, duplication, transfer and loss events are given in the inset, with the counts representing the number of distinct mappings giving the same outcome. All but the dark blue region of the parameter space suggest host shifts (transfer events). (B) Analysis based on the 42 regional isolates and their four nematode host species. Again, host shifts are predicted over the majority of the parameter space. Download FIG S3, TIFF file, 3.4 MB. Copyright © 2023 Papudeshi et al. 2023 Papudeshi et al. https://creativecommons.org/licenses/by/4.0/ This content is distributed under the terms of the Creative Commons Attribution 4.0 International license.

10.1128/mbio.00434-23.8 TABLE S5 Mixed-model analyses of variance for genetic similarity and gene flow as function of nematode host species and geographic distance. Along with Mantel tests of the correlation between genetic similarity and gene flow with distance at three spatial scales. Download Table S5, XLS file, 0.03 MB. Copyright © 2023 Papudeshi et al. 2023 Papudeshi et al. https://creativecommons.org/licenses/by/4.0/ This content is distributed under the terms of the Creative Commons Attribution 4.0 International license.

## DISCUSSION

Microbial symbionts often adapt and specialize to their hosts. And yet, numerous microbial species are characterized as host generalists, able to colonize and thrive in distinct host species. How do generalists evolve through time and space? Here, we examine the population genomics of the mutualist symbiont X. bovienii from a region where four nematode host species cooccur and compare them to globally available reference genomes. We find that, despite being associated with at least 10 nematode host species across the Northern hemisphere, X. bovienii forms a monophyletic group. Regionally, we found two distinct lineages of X. bovienii. One lineage was associated exclusively with a single nematode host species, while the other lineage was associated with three other nematode host species. Even though these two lineages were distinct and well supported, we detected recent gene flow across these lineages and among isolates from all four host species. Nevertheless, gene flow was higher if isolates shared a nematode host species and were collected from closer sites geographically. Thus, X. bovienii in this region can be viewed as a metapopulation, with gene flow tying this species together evolutionarily. Moreover, several genes were identified as being targets of differential selection within this population. The diverse functions of these genes, from insect toxins to antimicrobial effectors and resistance mechanisms, speak to the complex biotic environment imposing selection on these symbionts.

Xenorhabdus bacteria are specialized mutualists of nematodes, showing partial cocladogenesis with their hosts (26, 27); although this prior work suggested that X. bovienii could shift to distinct nematode host species, this conclusion was based on 11 allopatrically collected isolates and so could reflect few rare events. We sampled extensively from a sympatric population and predicted that the population structure of X. bovienii strains would mainly reflect their nematode host associations. We found only partial support for this hypothesis. For instance, nematode phylogeny presents S. affine and S. intermedium as sister taxa, equally distant from the sister taxa S. kraussei and S. texanum (Fig. 3B). However, the bacterial phylogeny based on core genes showed that S. affine-associated isolates were more closely related to isolates from S. kraussei and S. texanum than to those from S. intermedium. Furthermore, isolates associated with S. kraussei and S. texanum showed little structuring by nematode host in either the core or accessory genes (Fig. 3A and 4). These findings refute the hypothesis that S. bovienii consists of host-limited ecotypes (33). Rather, they suggest frequent host switching or recombination across isolates.

Based on the core phylogeny (Fig. 3A), successful host shifts have occurred in lineage 1, which includes isolates from three nematode hosts. For a host shift to occur, lineage 1 bacteria would be carried into an insect with one species of nematode and leave with another, and to persist, this novel pairing would have to outcompete the native pairs. In noncompetitive laboratory experiments, wherein aposymbiotic nematodes are paired with novel bacteria, S. affine nematodes were not able to accept X. bovienii bacteria from S. kraussei or S. texanum, while S. kraussei nematodes could accept S. affine-associated X. bovienii bacteria, albeit at such a severe fitness cost that the pairing would be unlikely to persist in nature (30, 34). In contrast, S. kraussei nematodes were found to accept S. texanum-associated X. bovienii with no reduction in fitness. These empirical results match the conclusion inferred from the phylogeny (Fig. 3) that host shifts across nematode clades occur less frequently than those within. Despite these findings, we found no genes significantly associated with S. affine in our genome-wide association study (GWAS) analysis. In fact, we found significant associations for only one nematode host, S. texanum. Association mapping in microbes is difficult due to high levels of linkage disequilibrium and population structuring (35, 36), and it is possible that treeWAS is overly conservative, as PopCOGenT detected selective sweeps associated with S. intermedium. One sweep occurred in the *mrx* fimbria region, which has been shown to be important in colonization of the nematode host (37). Additionally, the type 6 secretion system genes sweeping in this cluster could be important for interactions with the nematode host (38, 39). However, within lineage 1, few host-specific markers exist, suggesting that specificity may be due to multiple mechanisms or involve epistatic interactions, and therefore not be picked up in GWAS. In fact, different X. bovienii isolates from S. affine have shown distinct pathologies on nonnative nematodes (30, 40).

Despite the partial structuring by nematode host species, we found no gene flow discontinuity among our regional isolates (Fig. 5). In fact, high levels of gene flow were detected across some isolates associated with S. kraussei and S. texanum. Overall, observed recombination was higher when isolates shared a nematode host species and with geographic proximity (Fig. 7B), likely reflecting increased opportunities for genetic exchange and shared selective pressures. Each nematode host individual likely harbors a clonal population of X. bovienii (41, 42); however, to successfully invade and reproduce, several nematodes, which may carry different clones, must coinfect an insect host. Thus, it is within the insect that gene flow is likely to occur as distinct X. bovienii strains potentially interact with each other, with other Xenorhabdus species, and with the insect microbiome. Most clones were isolated within a few meters of each other, although some were found across study sites and, for one pair of global reference genomes, across continents (Fig. 7A). This pattern suggests that migration is important to the evolutionary history of X. bovienii. In most cases, migration will be local, driven by nematode movements, but longer-range migration could occur via erosion, predation of the insect host, or human agricultural activities. Regardless of the scale, migration has been implicated as a key factor facilitating gene sweeps through recombination (43).

Analysis of selective sweeps in the regional isolates of X. bovienii identified several genes (Tables 1 and 2) that are of known importance for entomopathogens (38). Specifically, nine toxin regions were found to be sweeping within the regional population. Two toxin genes were observed to be sweeping differentially across the clusters (Table S3). These sweeps may represent the ability to access additional insect species or to combat insect resistance (44). Additionally, 11 NRPS regions (3 differentially) were also found to be undergoing selective sweeps. These regions are important in the production of secondary metabolites, some of which are key in competition with the insect microbiota (45). Additionally, two antibiotic-related genes were found to be sweeping in cluster 2 and several multidrug transports, a type VI secretion system, and a siderophore in cluster 3, further establishing the dynamic competitive environment faced within the insect, as competition could come by attacking, resisting, or outgrowing a competitor (40). In fact, in cluster 2, which contains isolates from two nematode hosts, several genes were involved in amino acid and vitamin biosynthesis, which could reflect adaptations to better support nematode reproduction that would be beneficial across nematode species. Intriguingly, the successful experimental host shifts performed between S. kraussei and S. texanum (30) involved isolates from this population cluster, which leaves open the question of whether the successful host shift was facilitated by these recently shared genes. Future work in this system could examine the adaptive role of the identified sweeps and possible mechanisms of gene flow. Additionally, increased sampling coupled with additional experimental host shifts could help identify the basis of host specificity in this system.

Overall, our work supports the view that that gene flow in both the core and flexible genomes is important for maintaining the cohesiveness of X. bovienii across multiple nematode hosts. While our data suggest that host switching has occurred, it is less frequent than gene exchange, most likely due to the low fitness of newly associated pairs. This pattern contrasts with that found in the extensively studied S. aureus, which shows low levels of recombination in the core genome and frequent host switching, facilitated by acquiring host-specific genes from the host microbiome (17). The comparatively low microbial diversity in the insect host, coupled with more intense competition, may limit this pathway for host shifts in Xenorhabdus. In contrast, gene flow among coinfecting Xenorhabdus bacteria may allow beneficial alleles of genes, such as insect toxins or antimicrobials, to spread in response to local selection pressures. Thus, our results match findings in other systems that show local adaptation despite gene flow (46, 47) and differ from work that shows recombination barriers in sympatry (48, 49). Importantly, ours is one of only a few studies that examine the population structure and evolutionary history of a host-associated symbiont in a nonagricultural or medical setting, which increasingly enable the complex selective environments faced by microbes to become tangible.

## MATERIALS AND METHODS

### Study design.

Forty-two X. bovienii isolates associated with four distinct nematode host species were collected from three Indiana University Research and Training Preserve sites in Indiana, USA (Fig. 2). At each site, soil samples were collected and baited separately with insect hosts in the laboratory. Nematodes emerging from each soil-exposed insect were surface sterilized and crushed with a pestle to isolate their symbionts. The resulting supernatant was then plated onto NBTA (nutrient agar with 0.0025% bromothymol blue and 0.004% triphenyltetrazolium chloride), and bacterial colonies were streaked for isolation to create freezer stocks as previously described (50). Prior work showed little variation among bacterial symbionts within a nematode stock (41), and therefore, only one bacterial strain was selected per nematode stock for sequencing, with one exception, LD27A and LD27B, which were isolated from the same stock. Nematode species were identified using 28S and internal transcribed spacer (ITS) genes (51).

### Genome sequencing, assembly, and annotation.

Each X. bovienii isolate from a freezer stock was plated on NBTA, and a single colony picked for overnight culturing in LB medium (Difco). DNA extraction was performed following the DNeasy blood and tissue kit protocol for Gram-negative bacteria (Qiagen). Libraries with approximately 400-bp inserts were generated for each isolate and sequenced to generate paired-end reads on the Illumina NextSeq 500 platform using a 300-cycle kit. The reads were assembled with SPAdes assembler (52), and the contig statistics were assessed using QUAST version 5.0.2 (53). Additionally, 11 X. bovienii genomes (Fig. 2), four X. nematophila genomes, and four Photorhabdus genomes were downloaded from NCBI (Table S1). All 61 genomes were clustered by average nucleotide identity (ANI) using FastANI (54). The ANI results were plotted in R using the ggplot2 and Heatmap packages. Next, the protein-coding genes were predicted in all 61 genomes using prodigal version 2.6.3 (55), and the resulting genes were annotated using Prokka version 1.14.6 (56) against the Xenorhabdus gene database built from GenBank.

### Phylogenetic analysis.

Phylogenies were built from core regions. First, the assembled genomes were aligned using Mugsy version 1r2.3 (57). The core genome was defined as regions found in all 61 genomes that were greater than 3,000 bp in length and with less than 50% gaps (32). Trees were constructed using RAxML version 8.2.12 (58) using the general time reversible gamma (GTRGAMMA) model, with 100 bootstraps. Photorhabdus genomes were selected as the outgroup, and the resulting Newick tree was plotted using iTOL (59). These steps were repeated for just the 53 X. bovienii isolates, with X. bovienii CS03 as the reference genome. We repeated the analysis for just the 42 regional isolates, with MC081 as the reference genome to ensure that the order of the genes was represented with a local sample. To detect and account for recombination across X. bovienii isolates, which can bias phylogenetic inference, the core gene alignment and initial phylogenetic tree were further analyzed with ClonalFrameML version 1.12 (60). ClonalFrameML calculates the effect of recombination on the data set and generates a recombination-aware phylogenetic tree with adjusted branch lengths.

To compare the bacterial phylogeny to that of its nematode hosts, we used Parafit (61) via the ape R package (permute = 1,000, eigen value correction = Cailliez). Pairwise distances were calculated from each tree using the cophenetic.phylo ape function. A maximum-likelihood nematode phylogeny was constructed in MEGA (62) based on nematode 28S sequences available on GenBank (Table S1). We also conducted maximum-parsimony reconciliation via eMPRess to estimate host switching events (63).

### Pangenomic analysis.

To determine the flexible gene set across all the X. bovienii isolates, the genomes were run through a pangenomic analysis pipeline, Roary version 3.13.0 (64). Roary was run with the minimum sequence identity set to 90%, clustering protein coding genes from all 53 X. bovienii isolates. From the clustering results, the flexible and core gene sets were defined. To determine the grouping of X. bovienii isolates based on flexible gene sets, they were visualized using uniform manifold approximation and projection (UMAP) ordination plots in R.

### GWAS analysis.

To determine whether genetic markers could be associated with each nematode species, 53 X. bovienii genomes were run through treeWAS (36). The input to treeWAS was the recombination aware tree from ClonalFrameML and the core gene alignment used to build phylogenetic trees; we used the default parameters, setting the base *P* value to <0.05. In addition, all the SNPs were identified from the core gene alignment using SNP sites (65). TreeWAS was also run with the gene presence and absence table from pangenome analysis to identify flexible genes that were significantly associated with nematode hosts. The significant traits were annotated through tracing the location of the trait to the Prokka annotations output. To determine whether the results were dependent on using globally available Xenorhabdus genomes, we reran this analysis using just the 42 Indiana isolates.

### Gene flow analysis.

Recent gene transfer events across all X. bovienii genomes were identified using PopCOGenT (32). The assembled genomes were provided as input to PopCOGenT, which first identifies gene flow between each pair of genomes by identifying regions of higher-than-expected similarity (termed length bias) based on a null model of clonal descent (32). Then, genomes connected by gene flow are grouped into populations, and clusters within populations are defined by genomes sharing relatively higher gene flow between them (32). As this analysis showed that all of the Indiana isolates shared gene flow with two of the reference genomes, X. bovienii intermedium (isolated from SC, USA) and X. bovienii *kraussei Quebec* (isolated from Canada), falling into a single population group, we repeated this analysis for just the 42 isolates to examine gene flow events that could be potential targets of selection within the region. For each cluster, selection is inferred by PopCOGenT through determining events that share low nucleotide diversity within a cluster and have distinct mutations between clusters across both core and flexible regions. The resulting gene sweeps were annotated from the corresponding output from Prokka.

### Spatial analysis.

Geographic distance between isolates was based on previously established field transects (50) or calculated from coordinates. The three field sites were less than 28 km apart (Fig. 2), and within each field site, the isolates were collected less than 800 m apart from each other. Reference isolates were collected at least 370 km away from the Indiana isolates. We tested whether genetic similarity (average nucleotide identity [ANI]) and estimated gene flow (log_10_ length bias from the PopCoGenT analysis) were correlated with geographic distance (in log_10_ meters) by using Mantel tests via the vegan package in R. To test whether the nematode host species affected genetic similarity and gene flow, we classified each pair of isolates based on whether they were isolated from the same or different nematode species and then tested this effect in a full mixed-model analysis of covariance (ANCOVA), with geographic distance (in log_10_ meters) as a covariate and isolate identities and study sites as random effects. For each analysis, we tested all 53 X. bovienii isolates and then restricted the analysis to the 42 Indiana isolates, or to the isolates found at the MC and LD study sites.

### Data availability.

The genomes in this study have been deposited in GenBank under BioProject accession number PRJNA700777. In addition, the bioinformatics commands and files generated during analysis are available on GitHub (https://github.com/npbhavya/BovGenomes-analysis).
