# Supplementary figures and images for: Host Association and Spatial Proximity Shape but Do Not Constrain Population Structure in the Mutualistic Symbiont Xenorhabdus bovienii
Source: mBio. 2023 May 8;14(3):e00434-23. doi: 10.1128/mbio.00434-23 (PMC10306267; doi:10.1128/mbio.00434-23)

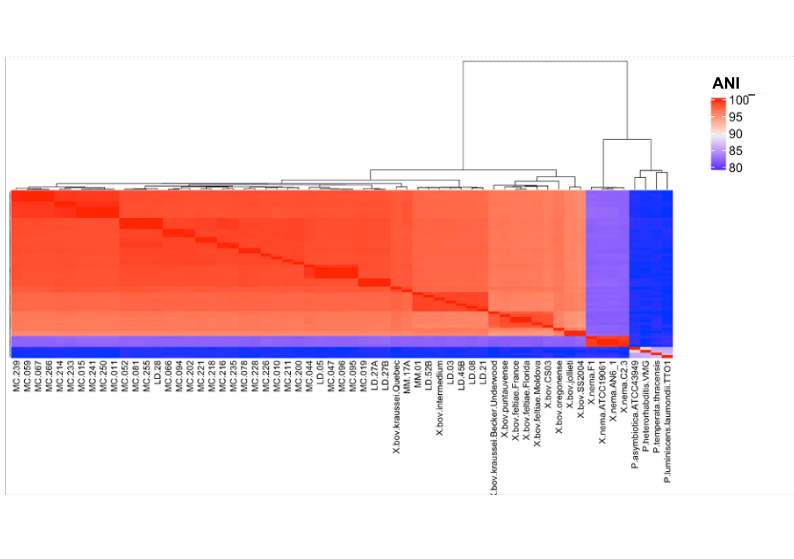

Supplement: FIG S1 [file mbio.00434-23-s0001.tiff]

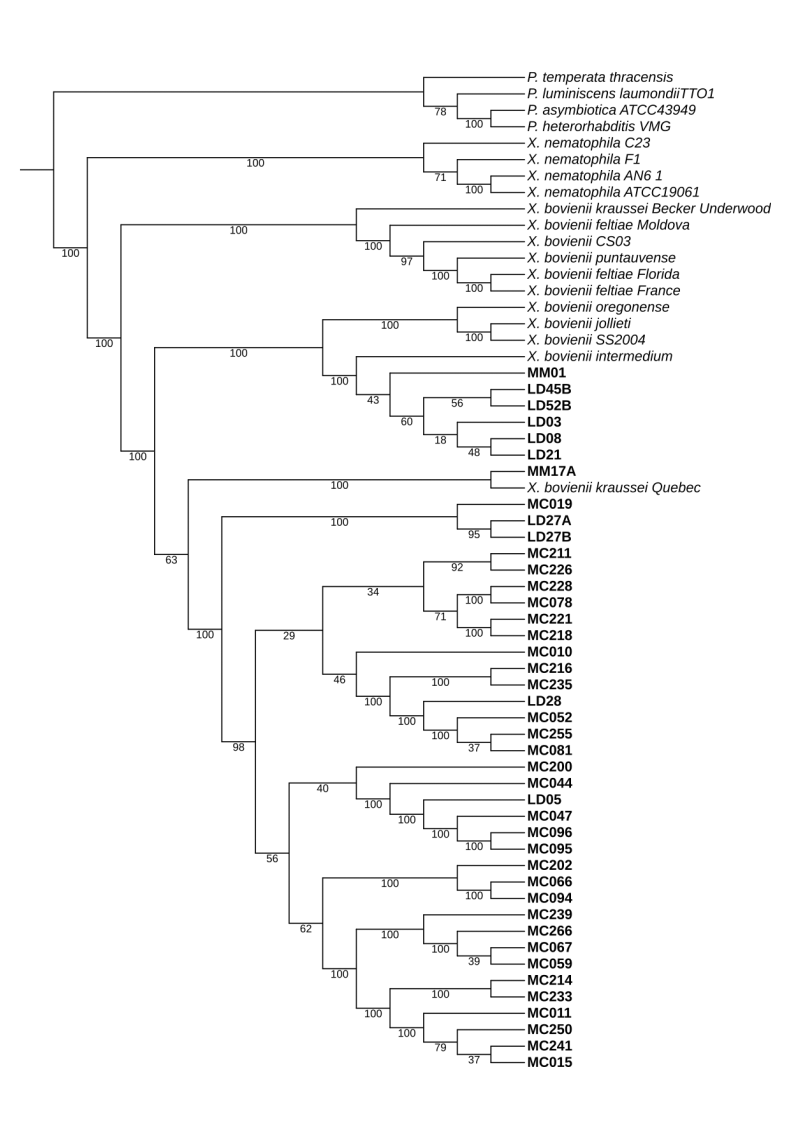

Supplement: FIG S2 [file mbio.00434-23-s0002.tiff]

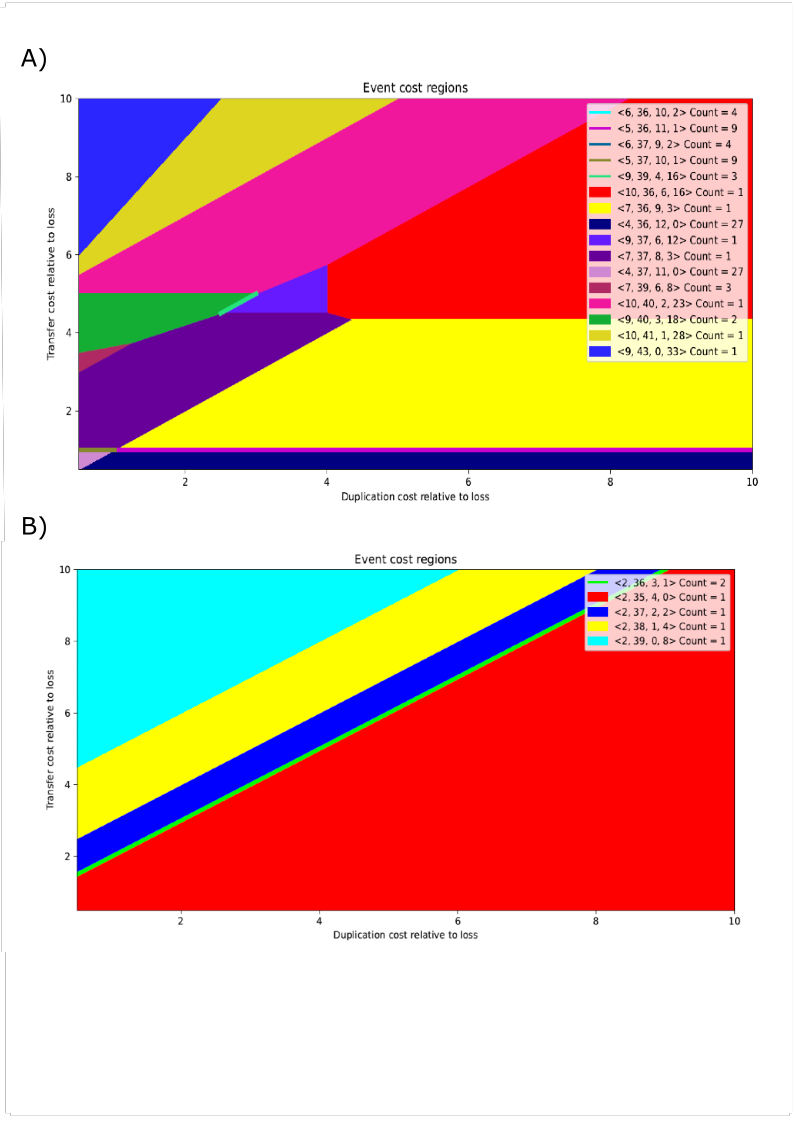

Supplement: FIG S3 [file mbio.00434-23-s0003.tiff]
