# Supplementary material for: Host Association and Spatial Proximity Shape but Do Not Constrain Population Structure in the Mutualistic Symbiont Xenorhabdus bovienii
Source: mBio. 2023 May 8;14(3):e00434-23. doi: 10.1128/mbio.00434-23 (PMC10306267; doi:10.1128/mbio.00434-23)
Supplement: TABLE S2 [file mbio.00434-23-s0005.docx]

| **Nematode host** | **Unique markers (Present in all members of each host and absent in all others)** | |
| --- | --- | --- |
|  | **Core gene set** | **Flexible gene set** |
|  | **(# SNPs)** | **(# genes)** |
| *S. affine* | 5, 13 | 0, 0 |
| *S. intermedium* |  |  |
| Including reference, | 22 | 47 |
| *X. bovienii intermedium* |  |  |
|  |  |  |
| Excluding reference | 73, 9132 | 5, 99 |
| *S. kraussei* |  |  |
| Including references: |  |  |
| *X. bovienii krausei Quebec,* | 0 | 0 |
| *X. bovienii Becker Underwood* |  |  |
|  |  |  |
| Including only referece: |  |  |
| *X. bovienii kraussei Quebec* | 0 | 1 |
|  |  |  |
| Excluding references | 0, 39 | 3, 18 |
| *S. texanum* | 0, 4 | 8*, 19* |
